# Evaluation of the Modified LIVestock SIMulator for Stall-Fed Dairy Cattle in the Tropics

**DOI:** 10.3390/ani10050816

**Published:** 2020-05-08

**Authors:** Christian A. Bateki, Uta Dickhoefer

**Affiliations:** Animal Nutrition and Rangeland Management in the Tropics and Subtropics, University of Hohenheim, 70599 Stuttgart, Germany; christian.bateki@uni-hohenheim.de

**Keywords:** cattle, (Sub-)Tropics, dry matter intake, model accuracy, ruminants, fiber

## Abstract

**Simple Summary:**

Models can play an important role in identifying and filling knowledge gaps related to sustainable resource use in (sub-)tropical livestock production systems. Yet, most simulation models used to study cattle production systems in the (Sub-)Tropics were developed using data that quantify and characterize biological processes of cattle kept in temperate regions, which may reduce the accuracy of predictions. Therefore, we adopted some published data that quantify and characterize biological processes of cattle kept in (sub-)tropical production systems to modify an existing ruminant livestock herd model. Then, the accuracy of predictions of feed intake and productive performance from the original and modified models were evaluated using meta data from (sub-)tropical stall-fed cattle. The modified model predicted voluntary dry matter intake and productive performance more accurately than the original model. Consequently, adopting relevant data that correctly describe the biological processes in (sub-)tropical cattle production systems is the way forward for improving simulation models for these systems.

**Abstract:**

Ruminant livestock systems in the (Sub-)Tropics differ from those in temperate areas. Yet, simulation models used to study resource use and productive performance in (sub-)tropical cattle production systems were mostly developed using data that quantify and characterize biological processes and their outcomes in cattle kept in temperate regions. Ergo, we selected the LIVestock SIMulator (LIVSIM) model, modified its cattle growth and lactation modules, adjusted the estimation of the animals’ metabolizable energy and protein requirements, and adopted a semi-mechanistic feed intake prediction model developed for (sub-)tropical stall-fed cattle. The original and modified LIVSIM were evaluated using a meta-dataset from stall-fed dairy cattle in Ethiopia, and the mean bias error (MBE), the root mean squared error of prediction (RMSEP), and the relative prediction error (RPE) were used to assess their accuracy. The modified LIVSIM provided more accurate predictions of voluntary dry matter intake, final body weights 140 days postpartum, and daily milk yields than the original LIVSIM, as shown by a lower MBE, RMSEP, and RPE. Therefore, using data that quantify and characterize biological processes from (sub-)tropical cattle production systems in simulation models used in the (Sub-)Tropics can considerably improve their accuracy.

## 1. Introduction

Tropical livestock production systems are changing quickly in response to drivers, such as growing human population and increasing urbanization, that cause a rise in the demand for food, especially of animal origin [1]. Hence, livestock farmers in these regions could enjoy access to new markets, diversify production, and thus improve their income security, if they take advantage of the demand-driven markets. Many (sub-)tropical livestock production systems are considered as inefficient due to the limited use of inputs and low product yields [2,3]. However, available resources (e.g., feed, land, and water) are limited, so that livestock farmers in these regions need to shift towards more efficient resource use and sustainable production to ensure future income and food security. Therefore, researchers need to holistically re-assess livestock systems in order to identify the best interventions for improving productivity and sustainability of livestock systems in the (Sub-)Tropics under the rapidly changing environmental and socio-economic conditions.

Simulation models have often been used [2,4] to enhance researchers’ knowledge on the feedforward and feedback interactions between different components (e.g., feed production, different animal herds, grazing land, and climate) of (sub-)tropical ruminant production systems. Such interactions are governed by biologically regulated processes (e.g., energy partitioning and use, feed intake regulation), which define the responses of ruminants to abiotic and biotic factors within a production system [5]. Ruminant production systems in the (Sub-)Tropics differ from those in temperate areas, for instance, in terms of the species or breeds of animals and their nutritional requirements [6,7], the availability and nutritional quality of feeds [8], and the prevalent environmental conditions. These differences should thus be correctly represented in simulation models to accurately evaluate resource use and productivity in ruminant production systems in the (Sub-)Tropics. However, most existing models (e.g., the LIVestock SIMulator (LIVSIM) and SAVANNA ecosystem models) used to simulate domestic ruminant herds in the (Sub-)Tropics and especially Sub-Saharan Africa reproduce biological processes based on data generated for ruminant production systems in temperate areas [9,10]. The present paper focuses on cattle, because they produce most of the meat and milk among all domestic ruminant species in the (Sub-)Tropics [11] and have thus been widely studied using simulation models.

Data to quantitatively characterize biological processes in (sub-)tropical cattle production systems are becoming increasingly available. Such data include estimates of the energy and protein requirements of cattle in the (Sub-)Tropics [6,7,12], their dry matter intake (DMI) capacity [13], and the nutritional composition of (sub-)tropical feedstuffs. We suggest that using such data in cattle simulation models for the (Sub-)Tropics could improve the accuracy with which feed resource use and productivity are simulated. Thus, the adjusted simulation models could serve as suitable tools to identify alternative feeding and management strategies for sustainable cattle production systems in the (Sub-)Tropics.

Building new simulation models is a more expensive and time-intensive process than adapting models already being used for (sub-)tropical cattle production systems [14]. As such, based on the review of five state-of-the-art models relevant for (sub-)tropical ruminant production systems [4], we selected the LIVSIM model for further modifications. The LIVSIM model was selected because it can predict feed resource use and performance of cattle and, unlike other models, also the associated environmental impacts of different feeding strategies [4]. The LIVSIM model is a dynamic model developed to assess the impacts of the allocation of feed resources on animal performance (i.e., productive and reproductive) in smallholder farming systems in Sub-Saharan Africa [15]. Using a monthly time step (i.e., 30.4 days), LIVSIM simulates cattle performance based on their genetic potential (i.e., breed-specific parameters) and the availability and nutritional quality of various feed resources. The energy and protein requirements of cattle in LIVSIM are estimated according to the recommendations of the Agricultural and Food Research Council (AFRC) [16] for dairy cattle. Lastly, LIVSIM can also simulate manure production and greenhouse gas emissions for different cattle production systems (e.g., confined dairy cows and/or free-ranging cattle).

Therefore, the present study aimed at modifying the predictions of some of the biological processes that govern cattle responses to feeding in LIVSIM and evaluating whether the modified model (hereafter called LIVSIM-mod) is more accurate in predicting voluntary DMI, final body weight (BW), and daily milk yields than the original LIVSIM. As such, we propose that modifying the simulation of biological processes in LIVSIM-mod based on data generated for (sub-)tropical cattle production systems will result in more accurate predictions of resource use and productive performance than from the original LIVSIM.

## 2. Material and Methods

### 2.1. Overview of the LIVSIM Model

**Figure 1:**
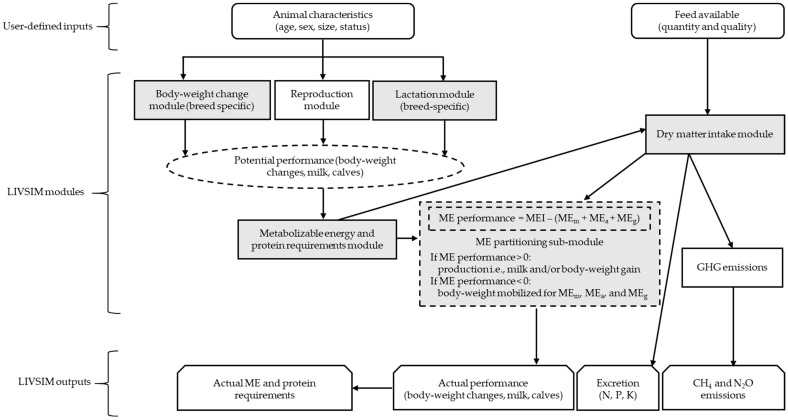
Schematic representation of the different components of the LIVestock SIMulator (LIVSIM) model. Grey boxes represent the modules in LIVSIM that were modified in the present study. Rectangular boxes with continuous lines represent LIVSIM modules and rectangular boxes with broken lines represent LIVSIM sub-modules. Rectangular boxes with smooth edges and trapezium-shaped boxes represent input and output components of the LIVSIM model, whereas the oval shape with broken lines contains the information being passed to the metabolizable energy and protein requirements module. ME performance: metabolizable energy available for productive performance, MEI: metabolizable energy intake, and ME_m_, ME_a_, and ME_g_: the metabolizable energy requirements for maintenance, activity, and gestation, respectively, when applicable (all in MJ/animal and day); GHG: greenhouse gas, N: nitrogen, P: phosphorus, K: potassium, CH_4_: methane, and N_2_O: nitrous oxide.

The LIVSIM model consists of three main components (Figure 1) including (i) a user-defined input component where details on the size and structure of the herd and related management decisions can be defined; (ii) various modules containing mathematical equations that describe different biological processes occurring in the animal; and (iii) an output component that summarizes the results from each simulation [10].

#### 2.1.1. LIVSIM Input Component

The user-defined input component includes data on herd and herd-related management options such as (i) breed- and sex-specific parameters such as life-time BW developments, pregnancy and lactation lengths, and milk fat and protein concentrations (g/kg milk) of the animal; (ii) herd size and structure characteristics—i.e., each animal in the herd is described by its breed, sex, age (years), BW (kg), and reproductive status (comprising of the stage of pregnancy (months) and calving interval (years) [10]; (iii) herd management rubrique, where users can define the target herd sizes and structure, culling ages for male and female animals, and maximum number of lactations per animal; (iv) available feed quantity (kg/animal and day) and nutritional quality; and (v) feeding strategies (i.e., daily quantity of different feeds allocated to animals in different herd classes). 

#### 2.1.2. LIVSIM Modules

The LIVSIM model contains five modules (i.e., square boxes with continuous lines, Figure 1), each describing a biological process. Detailed information on the equations used for each module is provided in [10,15]. In brief, the BW change module uses a simplified Brody model [17] to describe the potential BW gain of animals of different breeds and sexes [10,15]. The reproduction module controls conception in mature non-lactating and non-gestating females. Conception can be triggered either deterministically or stochastically based on the preference of the modeler. Deterministically, it is based on age and BW combinations [15], whereas, stochastically, it uses probabilities associated with the calving rate, postpartum length, presence or absence of a bull, and body condition [10]. Further, the lactation module describes the average daily milk yield throughout lactation for each breed based on the animal’s age and body condition [10]. The outputs from the BW change, reproduction, and lactation modules are then used to estimate the potential performance of animals in the herd.

Total metabolizable energy (ME) and metabolizable protein requirements of the animals are estimated from their potential performance according to [16] in the ME and protein requirements module and used to determine the required feed in the DMI module. This module uses the conceptual voluntary DMI model of [18], which was originally developed for high-performing dairy cows in temperate regions. The voluntary DMI (kg/animal and day) determines the total intakes of nitrogen, phosphorus, and potassium as well as of ME intake (MEI), which are then used to predict the actual performance (e.g., BW gain or loss and milk yield), greenhouse gas emissions, and nutrient excretion via urine and feces from the animal.

#### 2.1.3. LIVSIM Output Component

The output component of LIVSIM is a spreadsheet that summarizes the changes in BW, reproductive status, and the actual productive and reproductive performance of each animal in the herd for each time step (i.e., monthly) of a simulation. In addition, the nitrogen, phosphorus and potassium intakes, their excretion via urine and feces, their ME and metabolizable protein intakes, and the greenhouse gas emissions are reported on a monthly basis.

### 2.2. Modification of LIVSIM

Modifying the underlying data that characterize the biological processes governing cattle responses in LIVSIM could enable the model to better simulate dairy cattle production systems in the (Sub-)Tropics. Thus, a ME partitioning sub-module (i.e., grey square with broken lines in Figure 1) was created and integrated into LIVSIM-mod to determine the actual productive performance of the animals based on their actual DMI. In addition, four modules (i.e., grey squares with continuous lines in Figure 1) of the LIVSIM model were modified to simulate the biological processes they represent under (sub-)tropical husbandry conditions.

#### 2.2.1. Metabolizable Energy Partitioning Sub-Module

The ME partitioning sub-module (i.e., grey box with broken lines in Figure 1) represents a set of equations interacting with the DMI, BW change, and lactation modules to estimate the actual productive performance of the animals. The DMI module estimates the actual voluntary DMI (kg/animal and day), which is then used to derive the daily MEI as follows (Equation (1)):

(1) MEI=voluntary DMI × diet ME

where MEI is the metabolizable energy intake (MJ/animal and day), voluntary DMI the voluntary dry matter intake (kg/animal and day), and diet ME the dietary metabolizable energy concentration (MJ/kg dry matter).

To empirically establish the fraction of MEI available for milk production during lactation, average estimates were obtained from [19], who described MEI utilization in several dairy cattle breeds fed different diets. Accordingly, for diets containing concentrates, 100%, 88%, and 80% of the MEI available for performance (i.e., after accounting for the ME requirements for maintenance as well as activity and gestation requirements, if applicable) were used for milk production during early, mid, and late lactation, respectively. For diets without concentrates, 100%, 85%, and 74% of the MEI available for performance were used for milk production during early, mid, and late lactation, respectively. Furthermore, dairy cattle mainly mobilize BW for milk production during early lactation (i.e., first three months of lactation) [19]. Ergo, unlike in the original LIVSIM where BW mobilization could occur throughout lactation to maintain milk production, we limited BW mobilization for milk production to the first three months of lactation in LIVSIM-mod. If MEI is below the ME requirements for maintenance during early lactation, a maximum of 0.23% of the animal’s BW can be mobilized daily for either survival and/or milk production [20]. Beyond the first three months of lactation, milk production will cease, if MEI remains below the ME requirements for maintenance. In addition, if MEI is greater than the ME requirements for maintenance during early lactation, cows in LIVSIM-mod can mobilize BW for producing up to 60% of their potential milk yield predicted by the lactation module. Allowing for BW mobilization in LIVSIM-mod ensures that sufficient quantities of milk are available for calves when cows have a good body condition but their MEI is temporarily insufficient.

For the remaining phases of lactation, milk production is simply dependent on MEI available for performance as described for different diets above.

#### 2.2.2. Body Weight Change Module

Growth curves provide a mathematical description of the changes in BW of an animal from birth to when it attains its mature BW given adequate nutrition. In animal simulation models, growth curves are used to estimate the potential daily growth rate from which the nutritional requirements are estimated [21]. Several growth models are available [22,23] to describe growth patterns in cattle including the Brody model [17], the Gompertz model [24], and the von Bertalanffy model [25]. The original LIVSIM uses a simplified version of the Brody growth model [10] that assumes a constant potential exponential daily growth rate, which is independent of the animal’s BW until the age of puberty [26]. However, the Brody growth curve tends to over-estimate BW changes [10,27]. Thus, in an attempt to better mimic the potential daily growth rate for cattle in the (Sub-)Tropics, a parameterized Gompertz model [28] was implemented in LIVSIM-mod (Equation (2)). A potential advantage of the Gompertz curve is that it does not assume a constant potential growth for early phases of the animal’s growth as in the case of the Brody model, but assumes a growth proportional to the animal’s BW [26].

(2) $$\frac{\mathrm{dBW}}{\mathrm{dt}}={µ}_{0}\;\times \;\mathrm{BW}\;\times \;(\frac{\ln\;\times \;({\mathrm{BW}}_{f}\;/\;\mathrm{BW})}{\ln\;\times \;({\mathrm{BW}}_{f}\;/\;\mathrm{BW}0)})$$

where the first derivative of body-weight with respect to time dBW/dt is the potential daily body-weight growth rate (kg/day) [28], µ_0_ the Gompertz coefficient specifying the initial body-weight growth rate (0.015 per day for cattle) [28], BW the body-weight of the animal (kg), BW_f_ the body-weight (kg) at maturity (we assumed 90% of the maximum body-weight attainable, because under (sub-)tropical feeding conditions cattle rarely attain the genetic final body-weight [29]), and BW_0_ the body-weight (kg) of the animal at birth.

More so, to avoid unrealistic potential daily growth rates, the maximum daily growth was limited to 1.2 kg/animal as observed in an own (sub-)tropical meta-dataset [13]. The actual daily BW changes in LIVSIM-mod were then estimated (Section 2.2.5) based on the MEI (MJ/animal and day) available for BW gain or ME mobilized from body reserves when MEI was insufficient to sustain the ME requirements for maintenance and early lactation.

#### 2.2.3. Lactation Module

The lactation module in LIVSIM simulates the potential milk yield using lactation curves fitted from the literature data of the different breeds contained in the model. Lactation curves are used in animal simulation models to depict changes in daily milk yield from the time of parturition to the end of lactation [30], and thus to determine the potential milk yield. The predicted potential milk yield (kg/animal and day) is then modified by the age and body condition of the cow, as shown in [10]. The challenge with using this lactation module is that data must be obtained from the literature to fit the lactation curve for each new breed. In order to make the lactation module more generic and, at the same time, easy to parameterize, the lactation curve (Equation (3)) used in [9] was implemented in LIVSIM-mod, which requires mainly the peak milk yield (kg/animal and day) achieved during lactation and the month (n) in which the peak occurs.

(3) $$\mathrm{potential}\;\mathrm{milk}\;\mathrm{yield}=\frac{n}{\left(a\;\times \;\exp^{\left(k\cdot n\right)}\right)}$$

where potential milk yield is the potential milk yield during month n of the lactation cycle (kg/animal and day), n the time from calving (months), a the curve parameter, and k the shape parameter. The curve parameter (a) was calculated as [9]:

(4) $$a=\frac{1}{\left(\mathrm{peak}\;\mathrm{milk}\;\mathrm{yield}\;\times \;(1\;/\;T)\;\times \;\exp(1)\right)}$$

where peak milk yield is the peak milk yield during lactation (kg/animal and day) and T the month in which the peak milk yield is obtained. The shape parameter (k) was calculated as [9]:

(5) $$k=\frac{1}{T}$$

where T is the month in which the peak milk yield is obtained.

In the original LIVSIM, the final predicted potential daily milk yield was modified by the age and body condition factor of the cow [10]. This approach was therefore maintained when predicting the potential daily milk yield in LIVSIM-mod. The actual predicted milk yield (kg/animal and day) was, however, estimated based on the amount of the MEI available for lactation after ME partitioning.

#### 2.2.4. Metabolizable Energy and Protein Requirement Module

The recommendations for the ME and protein requirements used in the original LIVSIM were those according to the AFRC [16], estimated for dairy cattle in temperate production systems. To minimize potential errors in predicted cattle performance in (sub-)tropical systems, a modified factorial approach was used. This modified factorial approach builds on the German ME and protein requirement system (Gesellschaft für Ernährungsphysiologie (GfE) [31]) and the ME for growth requirement for cattle in warm areas of the French Institut National de la Recherche Agronomique (INRA) [32]. The GfE [31], the AFRC [16], and the INRA [32] ME recommendations all use a factorial approach to estimate the ME and protein requirements of cattle. However, the AFRC [16] and the INRA [32] systems require more dietary parameters (e.g., fermentable ME and metabolizability of the gross energy of the diet) than the GfE [31] system to match intakes and requirements of ME and protein in cattle. The fewer data requirements of the GfE [31] than the AFRC [16] and the INRA [31] system make it attractive for the (Sub-)Tropics, where detailed data on nutritional composition of diets are often lacking.

The ME requirements for each relevant metabolic function were calculated separately and then summed-up to yield the total ME requirements (MJ/animal and day), as shown in Equation (6):

(6) $$\mathrm{total}\;\mathrm{ME}\;\mathrm{requirement}={\mathrm{ME}}_{m}+{\mathrm{ME}}_{l}+{\mathrm{ME}}_{a}+{\mathrm{ME}}_{g}+{\mathrm{ME}}_{\mathrm{pf}}$$

where total ME requirement is the total metabolizable energy requirements for all metabolic functions (MJ/animal and day), and ME_m_, ME_l_, ME_a_, ME_g_, and ME_pf_ the metabolizable energy requirements for maintenance, lactation, activity, gestation, and protein and fat deposition (i.e., growth), respectively (all in MJ/animal and day).

The ME requirements (MJ/animal and day) for maintenance in cattle in temperate production systems differ from those of cattle in the (Sub-)Tropics [12]. As such, we estimated the ME requirements for maintenance (ME_m_) as (Equation (7)):

(7) $${\mathrm{ME}}_{m}=\mathrm{metabolic}\;\mathrm{body}\;\mathrm{weight}\;\times \;b$$

where metabolic body weight is the animal’s metabolic body-weight (kg^0.75^ body weight) and b the daily metabolizable energy requirements for maintenance per kg of metabolic body weight (MJ/kg^0.75^ body weight). For calves, b was 0.53 MJ/kg^0.75^ body weight [33] and for all other animals 0.631 MJ/kg^0.75^ body weight [12].

The potential ME requirements (MJ/animal and day) for lactation (ME_l_) were estimated according to [31] as (Equation (8)):

(8) $${\mathrm{ME}}_{l}=\mathrm{ME}\;\mathrm{for}\;\mathrm{milk}\;\times \;\mathrm{potential}\;\mathrm{milk}\;\mathrm{yield}$$

where ME for milk is the metabolizable energy requirement per kg of milk (MJ/kg milk) and the potential milk yield during month n of the lactation cycle in kg/animal and day (see Equation (3)). The ME requirements per kg of milk (ME for milk) were estimated as (Equation (9)):

(9) $$\mathrm{ME}\;\mathrm{for}\;\mathrm{milk}=\frac{(0.041\;\times \;\mathrm{milk}\;\mathrm{fat})+1.51}{k_{l}}$$

where milk fat is in g/kg milk and k_l_ the efficiency of ME use for lactation (i.e., 0.6 for *Bos taurus* × *Bos indicus* crossbreds and 0.53 for *Bos indicus* breeds [6]).

The ME requirements (MJ/animal and day) for activity (ME_a_) were estimated according to [16] (Equation (10)), because the GfE [31] provides no recommendations for estimating those requirements for cattle:

(10) $${\mathrm{ME}}_{a}=\frac{\left((2.6\;\times \;\mathrm{BW}\;\times \;\mathrm{horizontal}\;\mathrm{distance})\;/\;10^{6})+(28\;\times \;\mathrm{BW}\;\times \;\mathrm{vertical}\;\mathrm{distance})\;/\;10^{6})\right)}{k_{a}}$$

where 2.6 is the net energy (J) required to move 1 kg body-weight by 1 m horizontally (J/kg body weight and m), BW the body-weight (kg), horizontal distance is the horizontal distance (m) covered daily by the animal, 10^6^ a factor to convert the net energy required from J to MJ, 28 the net energy required to move 1 kg body-weight by 1 m vertically (J/kg body weight and m), vertical distance is the vertical distance covered daily by the animal (m), and k_a_ the efficiency of ME use for activity (0.7).

The ME requirements (MJ/animal and day) for gestation (ME_g_) were considered only for the last eight weeks of gestation and estimated as:

(11) $${\mathrm{ME}}_{g}=\frac{0.044\;\times \;\exp(0.0165\;\times \;\mathrm{gestation})}{k_{g}}$$

where gestation is the duration of gestation (days) and k_g_ is the efficiency of ME use for gestation (i.e., 0.2).

We adopted the ME requirements for growth recommended for cattle in warm areas by the INRA [32]. These energy and protein recommendations were not entirely adopted in LIVSIM-mod, because they require so many animal and feed parameters, making it quite impractical under typical (sub-)tropical husbandry conditions. Furthermore, the ME requirements per kg BW gain according to the AFRC [16], the GfE [31], and the INRA [32] are based on the animal’s current BW. Thus, these requirements assume that cattle are adequately fed and that their BW develops normally over time, as in most temperate systems. Yet, on-farm, animals in the (Sub-)Tropics often display retarded BW development relative to age, mainly due to poor breeding and undernutrition [34]. Thus, age-dependent ME requirements per kg BW gain were used in LIVSIM-mod to correctly simulate BW changes of cattle in the (Sub-)Tropics. First, the ME requirements according to the INRA [32] were regressed against the animals’ corresponding BW reported in the recommendations. Then, selected BW were replaced with cattle ages reported for *B. taurus* × *B. indicus* crossbreds and *B. indicus* breeds in different studies to obtain age-dependent ME requirements per kg BW gain (Table 1). Ergo, the resulting ME requirements per kg BW gain for animals older than two years in LIVSIM-mod were 27% higher than for those recommended by the INRA [31] to account for retarded or slower BW development observed under (sub-)tropical husbandry conditions.

The ME requirements (MJ/animal and day) for potential protein and fat deposition (ME_pf_; i.e., growth) were then estimated as (Equation (12)):

(12) $${\mathrm{ME}}_{\mathrm{pf}}=\mathrm{ME}\;\mathrm{for}\;\mathrm{gain}\;\times \frac{\mathrm{dBW}}{\mathrm{dt}}$$

where ME for gain is the metabolizable energy requirement per kg of body-weight gain at a given age (MJ/kg BW) and dBW/dt is the first derivative of body-weight with respect to time, which represents the potential daily body-weight growth rate (kg/animal and day).

The actual ME available for growth in LIVSIM-mod was determined by the MEI available for growth after ME partitioning. If MEI was less than ME requirements for maintenance, activity, and/or milk production in early lactation, net energy was mobilized from body reserves. The net energy was then used for different purposes (e.g., lactation) with an efficiency of 0.84 [31].

Similar to the ME requirements, metabolizable protein requirements (g/animal and day) of the AFRC [16] in the original LIVSIM were replaced by the utilizable crude protein (uCP) requirements (g/animal and day) according to the GfE [31] for each relevant metabolic function in LIVSIM-mod. The uCP is the sum of undegraded feed crude protein plus the microbial crude protein available in the duodenum of the cattle [35]. The metabolizable protein requirements differ from the uCP requirements in that the former is corrected for the proportion of true protein and its intestinal digestibility (i.e., truly digestible protein) while the latter is not (i.e., sum of the undegraded feed and microbial crude protein leaving the rumen). Thus, it is easier to estimate an animal’s protein requirements on a uCP basis using feed data commonly available in the (Sub-)Tropics than on a metabolizable protein basis.

The uCP requirements for each animal were calculated factorially, as shown in Equation (13).

(13) $$\mathrm{Total}\;\mathrm{uCP}\;\mathrm{requirement}={\mathrm{uCP}}_{m}+{\mathrm{uCP}}_{l}+{\mathrm{uCP}}_{g}+{\mathrm{uCP}}_{\mathrm{pf}}$$

where total uCP requirement is the total utilizable crude protein requirement (g/animal and day), and uCP_m_, uCP_l_, uCP_g_, and uCP_pf_ the utilizable crude protein required daily for maintenance, lactation, gestation, and protein and fat deposition (i.e., growth), respectively (g/animal and day).

The uCP requirements for each metabolic function considered in Equation (13) were estimated as reported in the GfE [31]. The net requirements of protein per kg BW in (sub-)tropical cattle reported by Valente et al. [36] rather than those suggested by the GfE [31] were used to estimate the uCP requirements for daily growth. Then, similar to the age-dependent ME recommendations for BW gain presented above, we developed an age-dependent net protein content per kg BW, as shown in Table 2.

Therefore, the uCP requirements (g/animal and day) for BW gain (uCP_g_) were estimated as:

(14) $${\mathrm{uCP}}_{g}=\mathrm{protein}\;\mathrm{content}\;\mathrm{in}\;\mathrm{gain}\;\times \;\frac{\mathrm{dBW}}{\mathrm{dt}}\;\times \;2.1$$

where protein content in gain is the protein content per kg body-weight gain at a particular age (Table 2), dBW/dt the first derivative of body-weight with respect to time which represents the potential daily body-weight growth rate (kg/animal and day), and 2.1 the efficiency of utilizing the utilizable crude protein for body protein accretion.

The actual uCP requirements for gain were then determined based on the actual daily BW changes as predicted from the MEI available for growth in the energy partitioning sub-module.

#### 2.2.5. Dry Matter Intake Module

Prediction of voluntary DMI in the original LIVSIM is based on the conceptual model of [18], developed for high-producing dairy cows in temperate regions. However, identifying a model that more accurately predicts voluntary DMI under (sub-)tropical feeding conditions has previously been recommended [10].

Bateki and Dickhoefer [13] identified and adjusted different conceptual models to adequately (i.e., accurately and precisely) predict voluntary DMI of stall-fed cattle in the (Sub-)Tropics. Consequently, the most adequate voluntary DMI prediction model identified by [13] for cattle under (sub-)tropical feeding conditions was adopted in LIVSIM-mod (Equation (15)):

(15) $$\mathrm{voluntary}\;\mathrm{DMI}=(\frac{\mathrm{NDF}\;\mathrm{intake}\;\mathrm{capacity}}{\mathrm{diet}\;\mathrm{NDF}\;}+\frac{\mathrm{total}\;\mathrm{ME}\;\mathrm{requirement}}{\mathrm{diet}\;\mathrm{ME}})\;/\;2$$

where voluntary DMI is the voluntary dry matter intake (kg/animal and day), NDF intake capacity the neutral detergent fiber intake capacity (kg/animal and day), diet NDF the neutral detergent fiber concentration in the diet (kg/kg dry matter), total ME requirement the total potential metabolizable energy requirements (MJ/animal and day) estimated according to Equation (6), diet ME the dietary metabolizable energy concentration (MJ/kg dry matter), and 2 to account for the blending of physiologically and physically regulated voluntary dry matter intake [13].

The NDF intake capacity (kg/animal and day) was calculated as:

(16) NDF intake capacity=0.0135 × BW

where BW is the animal’s body-weight (kg) and 0.0135 is the maximum daily amount of neutral detergent fiber (kg/kg BW) that can be consumed by lactating cattle in the (Sub-)Tropics.

The fact that voluntary DMI is estimated using the potential total ME requirements implies that the above intake model (Equation (15)) may over-estimate DMI. Potential productive performance of livestock is primarily driven by their genetic potential under optimum feeding conditions [37]. However, cattle in the (Sub-)Tropics are scarcely fed at optimum to fulfil their full productive genetic potential. Thus, to prevent over-estimation of the voluntary DMI in LIVSIM-mod, the predicted voluntary DMI estimate was multiplied by the BW condition index (Equation (17)):

(17) $$\mathrm{BW}\;\mathrm{condition}\;{\mathrm{index}}_{t}=\frac{\left({\mathrm{BW}}_{t}\;-\;{\mathrm{BW}}_{\min,t}\right)}{\left({\mathrm{BW}}_{\max,t}\;-\;{\mathrm{BW}}_{\min,t}\right)}$$

where BW condition index_t_ is the body-weight condition index at time t (kg/kg), BW_t_ the current body-weight of the animal of a given age at time t (kg), BW_min_,_t_ the minimum possible body-weight of an animal of a particular age at time t (kg), and BW_max,t_ the maximum body-weight attainable by an animal of a particular age at time t (kg). 

Similar to Equation (2), it was assumed that 90% of the maximum BW was attainable in LIVSIM-mod. The minimum and the maximum attainable BW were defined based on the literature data for each breed in the original LIVSIM [10].

### 2.3. Evaluation of LIVSIM-Mod

#### 2.3.1. Dataset Used for Model Evaluation

LIVSIM-mod was evaluated and compared to LIVSIM using data extracted from a study with stall-fed dairy cattle in Ethiopia [38], which evaluated the effect of lablab hay supplementation to 48 multiparous *B. indicus × B. taurus* cows fed forages from cereal–legume intercropping on feed voluntary DMI, apparent total tract digestibility, as well as on milk yield and milk composition. This study was selected because it lasted for 200 days (i.e., 60 days prepartum and 140 days postpartum), allowing for simulations over multiple time steps. For the present study, only data for the 140 days postpartum were used, because the average BW of the animals were not reported prepartum. Data (Table 3) for two dietary treatments (i.e., maize–lablab stover only and oats–vetch hay only fed ad libitum) out of the eight evaluated in [38] were chosen because of a lack of data on some dietary nutrient parameters (e.g., undegradable crude protein and fermentable ME of the diet) for the other six diets.

The original LIVSIM and LIVSIM-mod were parameterized using the animal and dietary parameters shown in Table 3 and run for five time steps (i.e., five months). The predicted final BW (kg/animal), voluntary DMI (kg/animal and day), and milk yield (kg/animal and day) from both LIVSIM versions were then compared with the corresponding data reported in [38].

#### 2.3.2. Statistical Evaluation

The original LIVSIM and LIVSIM-mod were evaluated for their accuracy in predicting the final BW, voluntary DMI, and daily milk yields. As first measure of accuracy, the mean bias error (MBE) in predicted final BW (kg/animal), voluntary DMI (kg/animal and day), and milk yields (kg/animal and day) was estimated as follows [39]:

(18) $$\mathrm{MBE}=\sum_{i}^{n}\frac{\left({\mathrm{observed}}_{i}\;-\;{\mathrm{predicted}}_{i}\right)}{q}$$

where MBE is the mean bias error, observed_i_ the observed value for either final body weight (kg), voluntary dry matter intake (kg/animal and day), or daily milk yield (kg/animal and day) for animal group i, predicted_i_ the predicted value for each of the parameters obtained from the both the original LIVSIM and LIVSIM-mod, respectively, for animal group i, and q is the number of pairs of the observed and predicted values for parameters being compared.

Further, to quantify the prediction error (i.e., measure of accuracy) associated with the final BW, voluntary DMI, and milk yield predictions obtained from both LIVSIM versions, the root mean squared error of prediction (RMSEP) was estimated as presented in [40]. Lastly, the RMSEP was expressed as a percentage of the observed mean value to obtain the relative prediction error (RPE) [40]. All statistical analyses were performed using the R software (version 3.4.0, the R foundation for Statistical Computing, Vienna, Austria).

## 3. Results

### 3.1. Voluntary Dry Matter Intake Predictions

The voluntary DMI predictions from the original LIVSIM for the maize–lablab and oats–vetch dietary treatments were greater than those from LIVSIM-mod and the measured values reported in the meta-data (Table 4).

The MBE (Table 5) in the voluntary DMI predictions from LIVSIM (−4.6 kg/animal and day) was greater than that in predictions from LIVSIM-mod (−0.2 kg/animal and day). The RMSEP and the RPE were also greater in the predictions from the original LIVSIM than those from LIVSIM-mod (Table 5).

### 3.2. Animal Productive Performance: Final Body Weight and Milk Yield Predictions

The final BW (kg/animal) predictions after 140 days postpartum by the original LIVSIM were greater than those by LIVSIM-mod (Table 4). The MBE in the predicted final BW was greater for LIVSIM (−49.5 kg/animal) than LIVSIM-mod (8.5 kg/animal) in the present study. Accordingly, the RMSEP and the RPE in predictions from the latter were lower than from the former. The milk yields (kg/animal and day) predicted by the original LIVSIM were also greater than the observed values in the meta-data. As in the case of the voluntary DMI and final BW, the MBE, the RMSEP, and the RPE were all lower for predictions from LIVSIM-mod than for those from the original LIVSIM (Table 6).

## 4. Discussion

LIVSIM-mod was obtained by modifying the cattle growth and lactation modules of LIVSIM, adjusting the estimation of the animals’ ME and protein requirements, and by adopting a feed intake prediction model developed for stall-fed cattle in the (Sub-)Tropics. LIVSIM-mod provided more accurate predictions of voluntary DMI of the animals, their final BW 140 days postpartum, and their daily milk yields than the original LIVSIM, as shown by the MBE, the RMSEP, and the RPE.

### 4.1. Accuracy of Models’ Prediction

The accuracy of a model’s predictions can be classified as excellent, good, fair, and poor, if the RPE is <10%, 10–20%, 21–30%, and >30%, respectively [41]. In the present study, the original LIVSIM predicted voluntary DMI and daily milk yield poorly, whereas the final BW predictions were good (Table 5 and Table 6). Meanwhile, the predicted voluntary DMI and final BW from LIVSIM-mod were excellent, and its predictions of daily milk yield were also good (Table 5 and Table 6). The accuracy of predicted animal performances from both LIVSIM versions resulted from the complex interactions between several processes including those needed to predict daily ME requirements, voluntary DMI, and ME partitioning [16,32,42]. Voluntary DMI is the most important determinant of productive and reproductive performance predictions in cattle simulation models [43]. The greater accuracy of LIVSIM-mod compared to the original LIVSIM in predicting voluntary DMI could be explained by two points. First, LIVSIM-mod employed the modified Mertens conceptual intake model [44], which was specifically adapted for stall-fed dairy cattle systems in the (Sub-)Tropics [13]. As such, the latter DMI model is more suitable for (sub-)tropical cattle production systems than the intake model of [18] used in the original LIVSIM, which was developed for high-producing dairy cows in temperate regions. Second, a BW condition index (i.e., Equation (17)) was used in LIVSIM-mod to down-scale the predicted DMI based on the body condition of the animal at each time step during simulations. The two conceptual models used in the present study predict DMI based on the theoretic potential (i.e., as defined by genetic potential) rather than actual (i.e., as defined by the animal’s status) productive performance of the animal. However, the potential productive performance of cattle kept in the (Sub-)Tropics is rarely achieved [37]. As such, using the estimated ME requirements for potential productive performance will lead to an over-estimation of DMI by any model.

Both the original LIVSIM and LIVSIM-mod over-estimated voluntary DMI (as shown by the negative MBE in Table 5). As such, in the original LIVSIM, both the final BW after 140 days postpartum and the daily milk yields were also over-estimated (−49.5 kg/animal and day and −5.3 kg/animal and day, respectively). In the case of LIVSIM-mod, only the daily milk yield was over-estimated (−0.4 kg/animal and day), whereas final BW after 140 days postpartum was under-estimated (8.5 kg/animal and day). The reason for the latter under-estimation is not clear but could probably be due to the ME partitioning during early lactation or the changes in ME requirements in LIVSIM-mod. In the present study, 100% of the MEI available for performance, and ME from mobilized body fat and protein (i.e., BW losses) was used to sustain up to 60% of the potential daily milk yield during early lactation [19]. In mid lactation (i.e., last 50 days of present simulation), 85% of the MEI available for performance were used for milk production and the remaining 15% MEI used for BW gain [19]. As such, all the animals lost BW in early lactation and regained some in mid lactation, although the BW gain predicted by LIVSIM-mod was lower than the observed gain during this phase. Since LIVSIM-mod at the same time over-estimated daily milk yields, the proportion of MEI used for milk production should be lower than the assumed 85%, increasing the proportion of MEI available for BW gain. Furthermore, the ME requirements per kg BW gain were greater by 27.5% in LIVSIM-mod than in the original LIVSIM. The greater ME requirements per kg BW gain additionally resulted in lower final BW predictions in LIVSIM-mod than in the original LIVSIM. Yet, overall, the changes made in LIVSIM-mod allowed for more accurate predictions of voluntary DMI and performance of cattle compared to the original LIVSIM.

### 4.2. Relevance of the Modifications Made in LIVSIM-Mod

Several livestock models exist that can be used to simulate cattle production systems in the (Sub-)Tropics, as shown by various reviews [4,45]. These livestock models differ in their assumptions and approach (e.g., steady state and dynamic) to simulate cattle production systems [45]. However, one of the challenges inherent to most livestock simulation models used in the (Sub-)Tropics is still the errors associated with predictions of resource use (i.e., feed intake) and productive performance (e.g., BW gain) [5,9]. Hence, improving the adequacy (i.e., accuracy and precision) of predictions should continue to receive attention to ensure that the basis on which conclusions will be drawn from simulation outcomes is reliable. Benchaar et al. [46] suggested that more accurate predictions from process-based models can be achieved by adjusting factors that regulate different biological processes. Thus, the present study leveraged existing data to characterize biological processes and modify how different processes are simulated for (sub-)tropical cattle production systems in order to enable LIVSIM-mod to better quantify the expected resource use and productive performance than the original LIVSIM. Moreover, fewer input data for dietary (e.g., acid-detergent insoluble nitrogen and fermentable ME) and animal (e.g., planes of nutrition to calculate ME requirement) parameters are required for LIVSIM-mod than for the original LIVSIM. Such lower input data requirements of LIVSIM-mod than of the original LIVSIM could enable users to easily parameterize and run it for (sub-)tropical cattle production systems.

### 4.3. Limitations of the Present Study

First, LIVSIM-mod should be applicable to a wide variety of ruminant production systems including small ruminants and crop–livestock cattle production systems. However, the present study used only data from stall-fed dairy cattle systems, implying that further evaluation is needed to ascertain how well LIVSIM-mod simulates other husbandry systems such as those mentioned above. Second, the present study could not validate LIVSIM-mod due to a lack of access to suitable data. For instance, the number of mean observations of the meta-dataset used in the study were not enough to distinguish systematic errors from random errors associated with model predictions [47]. Further, the dataset used for the present evaluation is small and focuses only on a short time period. As such, it was not possible to assess how the modifications in LIVSIM-mod may affect the adequacy of predictions for different groups of animals (e.g., heifers and calves), the reproductive performance, or the overall development in the size and structure of cattle herds over time. Therefore, there is still a need for validating LIVSIM-mod using a more comprehensive dataset from the (Sub-)Tropics.

## 5. Conclusions

LIVSIM-mod predicts feed resource use and productive performance of stall-fed dairy cattle in the (Sub-)Tropics more accurately than the original LIVSIM, as it accounts for differences in voluntary feed intake capacity, growth and lactation curves, and ME requirements for maintenance and BW gain of cattle between temperate and (sub-)tropical husbandry systems. It may thus be a useful tool to assess strategies for improving productivity and sustainability of stall-fed dairy cattle systems in the (Sub-)Tropics. Further research should assess how well LIVSIM-mod can simulate resource use and productive and reproductive performance of other cattle groups (e.g., calves and heifers) in diverse production systems in the (Sub-)Tropics.

## Figures and Tables

**Table 1:** Net energy requirements per kg of body-weight change (MJ/kg body-weight; k_pf_ = 0.4 ^1^) for female and male zebu cattle and crossbred cattle of different ages in the (Sub-)Tropics.

Sex	Species	Age (Years)						
		0.0	0.1	1.5	3.0	4.5	5.5	20.0
Female	*Bos indicus × Bos taurus*	3.4	4.0	9.6	16.5	23.4	23.4	23.4
	*Bos indicus*	3.4	3.9	8.6	12.9	16.2	17.0	17.0
Male	*Bos indicus × Bos taurus*	3.4	4.0	9.1	15.5	21.8	25.1	25.1
	*Bos indicus*	3.4	3.9	7.9	11.9	14.8	18.8	18.8

^1^ k_pf_ is the efficiency of utilization of metabolizable energy for protein and fat deposition [31].

**Table 2:** Net requirement of protein per kg of body-weight gain for female and male zebu and crossbred cattle of different ages in the (Sub-)Tropics.

Sex	Species	Age (Years)						
		0.0	0.1	1.5	3.0	4.5	5.5	20.0
Female	*Bos indicus × Bos taurus*	236	182	174	171	170	170	170
	*Bos indicus*	236	182	173	170	168	168	168
Male	*Bos indicus × Bos taurus*	236	182	175	172	170	169	169
	*Bos indicus*	236	182	174	168	167	167	167

**Table 3:** Animal and dietary parameters from stall-fed dairy cattle in Ethiopia used for parameterizing and evaluating LIVSIM-mod.

Parameter	Treatment	
	Maize–Lablab	Oats–Vetch
*Animal*		
Number of cows	6	6
Age ^1^, years	5.2	5.2
Body weight at calving, kg	415	432
Body weight at 140 days postpartum, kg	386	399
Voluntary dry matter intake, kg/animal and day	9.8	9.7
Lactation length, months	10	10
Peak milk yield ^2^, kg/animal and day	20	20
Month of peak milk yield ^2^	2	2
Milk yield, kg/animal and day	8.25	6.82
Milk fat, g/kg milk	46.3	46.6
Milk protein, g/kg milk	29.0	31.2
*Diet*		
DM, g/kg as fed	890	888
CP, g/kg DM	93.1	87.5
Undegradable CP ^3^, g/kg CP	250	220
Acid detergent insoluble nitrogen ^3^, g/kg DM	0.9	1.1
CP fraction a ^4^	0.24	0.18
CP fraction b ^5^	0.57	0.57
CP fraction c ^6^	0.04	0.05
Neutral detergent fiber, g/kg DM	550	610
Gross energy ^3^, MJ/kg DM	17.2	17.6
Metabolizable energy, MJ/kg DM	9.4	9.6
Fermentable metabolizable energy of diet ^3^, MJ/kg DM	7.2	7.4
DM digestibility, g/kg DM	659	683
Phosphorus, g/kg DM	4.5	3.6
Potassium, g/kg DM	25.0	29.8

CP, crude protein; DM, dry matter. ^1^ Not reported but estimated based on the average (i.e., 2–4) number of parity reported in the study; ^2^ adopted from [9] to parameterize the lactation curve; ^3^ obtained from own data and [16]; ^4^ the proportion of water-soluble nitrogen in total nitrogen of the diet, ^5^ the proportion of potentially degradable nitrogen other than water-soluble nitrogen of the diet, and ^6^ fractional rumen degradation rate per hour of the b fraction of feed nitrogen.

**Table 4:** Observed and predicted values from different LIVSIM versions for voluntary dry matter intake (DMI) and productive performance of stall-fed dairy cows in Ethiopia.

Experimental Diet	Parameter								
	Voluntary DMI (Kg/Animal and Day)			Final Body Weight (Kg/Animal)			Milk Yield (Kg/Animal and Day)		
	*Observed*	*LIVSIM*	*LIVSIM* -*Mod*	*Observed*	*LIVSIM*	*LIVSIM* *-Mod*	*Observed*	*LIVSIM*	*LIVSIM* *-Mod*
Maize-lablab	9.8	14.4	10.2	386	454	385	8.3	13.1	8.1
Oats-vetch	9.7	14.2	9.6	399	430	383	6.8	12.6	7.8

**Table 5:** Statistical evaluation of different LIVSIM versions for predicting voluntary dry matter intake in stall-fed dairy cattle in Ethiopia.

Statistical Measure	LIVSIM	LIVSIM-Mod
MBE ^†^, kg/animal and day	−4.6	−0.2
RMSEP ^††^, kg/animal and day	4.6	0.3
RPE ^†††^, % mean observed value	47.0	3.0

^†^ MBE = mean bias error; ^††^ RMSEP = root mean squared error of prediction; ^†††^ RPE = relative prediction error.

**Table 6:** Statistical evaluation of different LIVSIM versions for predicting final body weight (kg/animal) after 140 days postpartum and milk yield (kg/animal and day) in stall-fed dairy cattle in Ethiopia.

Statistical Measure	Predicted Parameter			
	Final Body Weight		Daily Milk Yield	
	LIVSIM	LIVSIM-Mod	LIVSIM	LIVSIM-Mod
MBE ^†^	−49.5	8.5	−5.3	−0.4
RMSEP ^††^	52.8	11.3	5.3	0.7
RPE ^†††^, % mean observed value	13.5	2.9	70.5	10.0

^†^ MBE = mean bias error; ^††^ RMSEP = root mean squared error of prediction; ^†††^ RPE = relative prediction error.
